# Utilization of Cobalt and its Oxide/Hydroxide Mediated by Ionic Liquids/Deep Eutectic Solvents as Catalysts in Water Splitting

**DOI:** 10.1002/open.202400136

**Published:** 2024-08-30

**Authors:** Chenyun Zhang, Jie Wang, Jianjiao Jin, Jiahao Wang, Te Bai, Jiacheng Xu, Shun Wang, Lihua Xu, Jing Zhang

**Affiliations:** ^1^ Wuxi Vocational Institute of Arts & Technology Yixing Jiangsu 214200 China; ^2^ Kaishi Faurecia Aftertreatment Control Technologies Co., Ltd Wuxi Jiangsu 214000 China; ^3^ Shazhou Professional Institute of Technology Zhangjiagang Jiangsu 215600 China; ^4^ Wuxi Vocational College of Science and Technology Wuxi Jiangsu 214028 China

**Keywords:** Ionic liquids, Deep eutectic solvents, Cobalt and its oxide/hydroxide, Water splitting, Hydrogen evolution reaction, Oxygen evolution reaction

## Abstract

With the ever‐growing global demand for sustainable energy solutions, hydrogen has garnered significant attention as a clean, efficient, and renewable energy source. In the field of hydrogen production, catalyst research stands out as one of the foremost areas of focus. In recent years, the preparation of electrocatalysts using ionic liquids (ILs) and deep eutectic solvents (DESs) has attracted widespread attention. ILs and DESs possess unique physicochemical properties and are recognized as green media as well as functional materials. Cobalt‐based catalysts have proven to be efficient electrocatalysts for water splitting. Incorporating ILs or DESs into the preparation of cobalt‐based catalysts offers a remarkable advantage by allowing precise control over their structural design and composition. This control directly influences the adsorption properties of the catalyst's surface and the stability of reaction intermediates, thereby enabling enhanced control over reaction pathways and product selectivity. Consequently, the catalytic activity and stability of cobalt‐based catalysts can be effectively improved. In the process of preparing cobalt‐based catalysts, ILs and DESs can serve as solvents and templates. Owing to the good solubility of ILs and DESs, they can efficiently dissolve raw materials and provide a special nucleation and growth environment, obtaining catalysts with novel‐structures. The main focus of this review is to provide a detailed introduction to metal cobalt and its oxide/hydroxide derivatives in the field of water splitting, with a particular emphasis on the research progress achieved through the utilization of IL and DES. The aim is to assist readers in designing and synthesizing novel and high‐performance electrochemical catalysts.

## Graphical abstract

Cobalt‐based catalysts have gained significant attention in the field of water splitting. By employing ILs and DESs in the synthesis of cobalt‐based catalysts, it is possible to tailor their structures and compositions, thereby influencing the adsorption properties of the catalyst surface and stability of reaction intermediates. This synthetic strategy enables manipulation of the reaction pathway and product selectivity, leading to enhanced catalytic activity and stability. This review primarily focuses on the research progress achieved through the application of ILs and DESs in the study of metal cobalt and its oxide/hydroxide derivatives in water splitting.

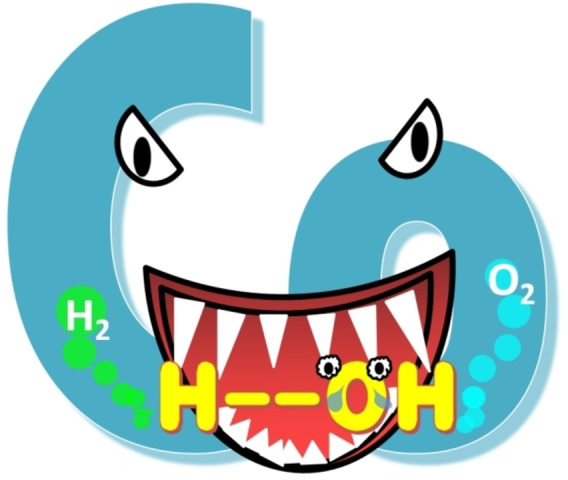

## 1 Introduction

**Figure 1:**
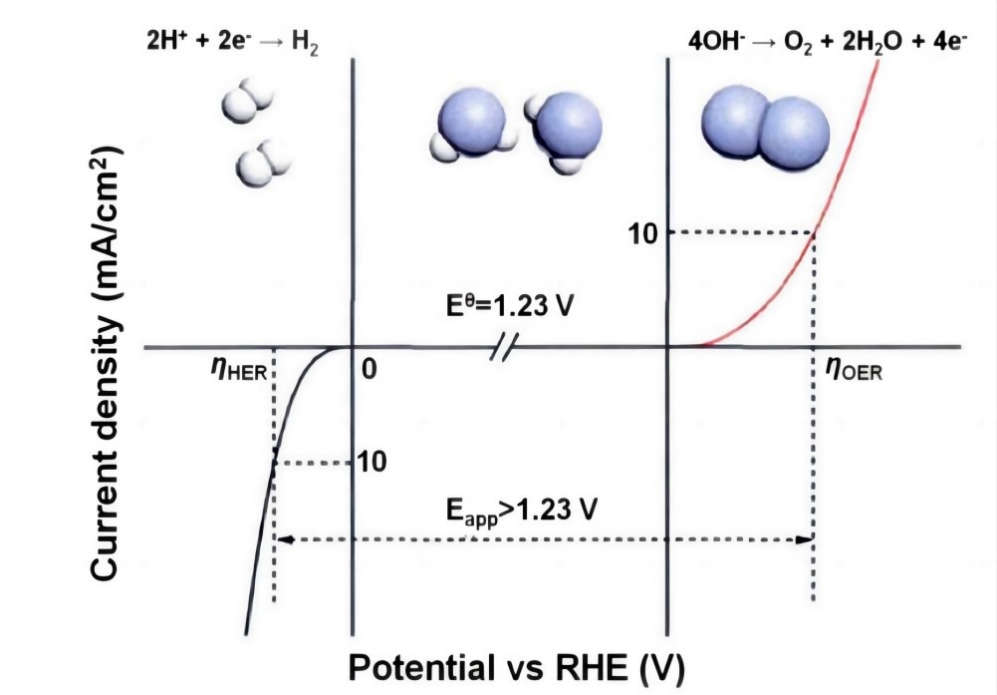
Comparison of theoretical decomposition potential and actual decomposition potential in water splitting. Reprinted with permission from ref. [6], copyright 2017 wiley.

With the increasing severity of the global fossil energy crisis and environmental pollution, countries around the world are intensifying their research of new energy technologies.^[1]^ Hydrogen, with its high energy density and clean combustion product (H_2_O), is considered as one of the most ideal clean energy sources.[^2^, ^3^] Water splitting is a common hydrogen production technology which the anode undergoes the oxygen evolution reaction (OER), while the cathode experiences the hydrogen evolution reaction (HER).[^4^, ^5^] The theoretical potential required to drive the entire reaction is 1.23 V. However, in practical applications, higher potentials are often needed to overcome various energy losses and electrode polarization phenomena during the reaction process, as shown in Figure 1.^[6]^

Therefore, catalysts play a crucial role in reducing the overpotential of water splitting.[^7^, ^8^] Currently, precious metal catalysts like platinum and palladium are commonly used for this purpose. However, their high cost and limited availability make it important to explore alternative transition metal catalysts that are more affordable and abundant. Cobalt and cobalt‐based oxides/hydroxides as catalysts are one of the hot research topics in the field of water splitting. In HER, a metal catalyst's ability to adsorb hydrogen is determined by the adsorption Gibbs free energy (ΔG_H_*). A smaller absolute value of ΔG_H_* indicates stronger hydrogen adsorption capacity and better performance in HER.[^9^, ^10^] Cobalt exhibits a small ΔG_H_* value, highlighting its strong hydrogen adsorption capability and favorable performance in HER. In OER, the interaction between cobalt‘s 3d orbital electrons and oxygen functional groups helps regulate the bonding strength of Co−OH, thereby greatly enhancing its catalytic performance.

Although Cobalt and cobalt‐based oxides/hydroxides show great promise in water splitting, there is still a certain gap compared to precious metal electrocatalysts. Therefore, researchers have been exploring various strategies to enhance their efficiency and stability by altering their morphology and electronic structure. One effective approach is the utilization of novel media or reactants. In recent years, significant progress has been made in preparing cobalt‐based electrocatalysts using ionic liquids (ILs) and deep eutectic solvents (DESs).

ILs are salts composed of cations and anions with a large difference in volume.[^11^, ^12^, ^13^] The asymmetry in the size of the cations and anions makes it difficult for them to pack densely or crystallize. Therefore, ILs have low melting points, usually below 100 °C. ILs are typically composed of organic cations and inorganic or organic anions. Common cations include quaternary ammonium ions, quaternary phosphonium ions, and imidazolium ions, while anions include halide ions, tetrafluoroborate ions, and hexafluoride ions. The structural characteristics of ILs lie in the interactions between the cations and anions, which include Coulombic forces, hydrogen bonding, van der Waals forces, and so on. The diversity of cation‐anion combinations in ILs leads to their designability. These structures determine their unique physical and chemical properties, such as low vapor pressure, high thermal stability, and good conductivity. They exhibit great application potential in chemical reactions as solvents or catalysts, as well as in energy storage, green chemical synthesis, biomedicine, and other fields. In the synthesis of transition metal catalysts, the use of ILs as solvents or templates has the following advantages:

1. Enhancing solubility: ILs exhibit high solubility for a wide variety of inorganic and organic compounds and disperse active species uniformly, thereby obtaining favorable catalyst morphologies and improving catalytic performance.
2. Regulating reaction mechanism: The unique properties of ILs lead to special reaction mechanisms, effectively controlling the nucleation and growth mechanisms, crystal structure, and electronic properties of cobalt‐based catalysts, thus regulating catalytic activity and selectivity.
3. Stabilizating intermediates: The high polarity and strong interactions of ILs can effectively stabilize intermediates during catalyst synthesis. Additionally, owing to their high coordination ability, ILs can form stable complexes with transition metal ions. This stability reduces side reactions and minimizes unnecessary decomposition or deactivation.

Since their discovery in 1914, ILs have been extensively researched in the fields of chemistry and materials science. However, ILs have limitations such as high cost, difficulties in purification, poor biocompatibility, and low degradation rate. Moreover, concerns regarding the safety of ILs have arisen.^[15]^ In this context, DESs have emerged as a promising alternative to ILs. DESs are composed of two or three components, hydrogen bond acceptors (HBAs) and hydrogen bond donors (HBDs), in a specific stoichiometric ratio to form a eutectic mixture.^[16]^ The structural characteristics of DESs primarily lie in the formation of their hydrogen bond networks. At appropriate molar ratios, a large number of hydrogen bonds are formed between HBAs and HBDs. The special structures of DESs lowers their freezing point compared to their individual components, making them typically liquid at room temperature. Taking the example of a DES composed of choline chloride (ChCl) and urea in a molar ratio of 1 : 2, its freezing point is around 12 °C, which is approximately 200 °C lower than the individual components alone.[^17^, ^18^, ^19^]

Although ILs and DESs differ in structure and properties, they are both substances maintained in a liquid state by specific intermolecular forces. ILs maintain their liquid state primarily through charge interactions, while DESs maintain their liquid state through weak interactions such as hydrogen bonding. DESs share similar properties to ILs, such as high stability, wide electrochemical window, and low volatility.^[20]^ Both ILs and DESs can adjust their properties by changing the types and ratios of their components, thus making them interrelated in some aspects. However, DESs have the following advantages over ILs:

1. Lower cost and higher availability: DESs are typically composed of common compounds, resulting in a lower production cost and easier availability compared to ILs. ILs often require more expensive raw materials and complex synthesis methods for their preparation.
2. Easy purification and recovery: DESs can be easily purified and recovered through simple methods, such as distillation or extraction. In contrast, the purification and recovery of ILs are relatively more challenging and often require more complex techniques.
3. Good biocompatibility and degradability: DESs generally exhibit better characteristics in terms of biocompatibility and degradability compared to ILs, making them more acceptable for various biologically‐related applications.

Since their discovery in 2003, DESs have quickly gained attention and found applications in diverse fields such as organic synthesis, separation techniques, energy conversion, nanomaterial preparation and so on. The extensive range of applications highlights the promising prospects for DESs in the future.^[21]^ Particularly in the synthesis of transition metal catalysts, DESs have demonstrated comparable or even superior performance compared to ILs. This further emphasizes the potential of DESs as effective alternatives and opens up new possibilities for catalyst design and development.

ILs and DESs, recognized as green media and functional materials, possess unique liquid environments and supramolecular structures. They are widely used as solvents and templates in modifying the structure and morphology of cobalt and its oxides/hydroxides, thereby enhancing catalytic performance. This review, combined with the efforts of our research group, offers an in‐depth overview of the synthesis of cobalt‐based electrolytic water splitting facilitated by the involvement of ILs and DESs (Table 1). This review is primarily divided into three sections. The first part introduces the research background of preparing cobalt‐based catalysts using ILs and DESs and the advantages of involving them in the preparation of electrocatalysts. The second part discusses in detail the application of ILs and DESs in the preparation of cobalt‐based metal, oxide, and hydroxide catalysts. The third part summarizes the key findings of the review and provides a perspective on the future development of cobalt‐based catalysts based on ILs and DESs.

**Table 1:** Summarizes the performance of cobalt‐based catalysts based on ILs/DESs in terms of HER, OER, and water splitting in this study.

Catalyst	Applied IL/DES	Preparation method	Catalytic performance								Ref.
			HER			OER			Overall water splitting		
			Electrolyte	*η*(mV)@Current density (mA cm^−2^)	Tafel slope (mV dec^−1^)	Electrolyte	*η*(mV)@Current density (mA cm^−2^)	Tafel slope (mV dec^−1^)	Electrolyte	Potential (V)@ Current density (mA cm^−2^)	
Co	ChCl/malonic acid	Electrodeposition	–	–		1 M KOH	350@10	76	–	–	[25]
Co@NPC	ChCl/urea/glucose acid	Calcination	0.5 M H_2_SO_4_	215@10	70	–	–	–	–	–	[26]
Co@NPC	ChCl/urea/glucose acid	Calcination	1 M KOH	274@10	91	1 M KOH	430@10	87	1 M KOH	1.74	[26]
Ni−Co‐Sn	ChCl/EG	Electrodeposition	1 M KOH	–	121	–	–	–	–	–	[28]
Fe‐Co	ChCl/urea	Electrodeposition	0.5 M NaOH	89.2@10	44.6	–	–	–	–	–	[29]
N,S‐NiCoFe	FeCl_3_ ⋅ 6H_2_O/CoCl_2_ ⋅ 6H_2_O/NiCl_2_ ⋅ 6H_2_O/L‐cysteine	Calcination	–	–	–	1 M KOH	251@10	58	–	–	[30]
CoO	[BMIM]Tf_2_N	Calcination	–	–	–	1 M KOH	320@10	70	–	–	[30]
NiCo_2_O_4_	ChCl/glycerol	1.Co‐precipitation method 2.Calcination	–	–	–	1 M KOH	320@10	61	–	–	[31]
CoV_2_O_6_	ChCl/malonic acid	Calcination	–	–	–	1 M KOH	324@10	–	–	–	[38]
CoFe	[PMIM]BF_4_	Solvothermal method	–	–	–	1 M KOH	350@10	57.5	–	–	[32]
CoNi	[PMIM]BF_4_	Solvothermal method	–	–	–	1 M KOH	420@10	98.7	–	–	[32]
CoFe‐LDH	ChCl/urea	Water injection method	–	–	–	0.5 M KOH	–	–	–	–	[40]

## 2 The Preparation of Cobalt and its Oxide/hydroxide Catalysts for Water Splitting Involving the Participation of ILs and DESs

### 2.1 Cobalt‐Based Metal Catalysts Based on ILs and DESs

The metal cobalt has a high electrical conductivity, which is beneficial for the transfer of electrons and ions, facilitating the easy transfer of charges generated in the electrolysis reaction. Cobalt atoms have unfilled d‐electron orbitals, allowing them to interact with reaction intermediates and influence their structure and electronic properties, thereby improving the activity and efficiency of catalytic reactions. In addition, the surface of cobalt metal has abundant reactive sites that can provide active centers required for adsorption and catalytic reactions, thereby improving reaction rate and selectivity. By selecting suitable synthesis methods and controlling conditions, it is possible to regulate and optimize the morphology, lattice structure, and size of cobalt metal, thereby controlling its catalytic performance. Furthermore, Co metal shows good durability in water splitting, allowing for stable and long‐lasting catalysis of oxygen and hydrogen generation. Consequently, Co has garnered significant interest as a crucial catalyst for water electrolysis.[^22^, ^23^, ^24^]

Renjith et al.^[25]^ applied ChCl/malonic acid DES as an electrolyte and successfully modified the electrode surface with cobalt nanoparticle cluster thin films through a one‐step electrodeposition method for OER. This method overcame the cumbersome reaction steps involved in modifying the electrode with nanoparticle clusters, including chemical synthesis, purification, and embedding onto the electrode surface. In addition, the high viscosity of DES played a crucial role in this process, slowing down the transfer rate of conductive particles. As a result, the morphology of the deposited products changed. This is a significant advantage as it provides more control over the morphology and structure of the deposited films, which can affect their catalytic performance. This approach successfully achieved fine deposition of Co metal, forming dendritic or dense aggregated films. In 1 M KOH, the catalyst exhibited an overpotential of 350 mV at a current density of 10 mA cm^−2^ and a Tafel slope of 76 mV dec^−1^. DES not only provides a novel platform for electrocatalytic reactions but also demonstrates how to optimize the electrode modification process by adjusting the physicochemical properties of the electrolyte. This study offers a new perspective for designing innovative electrocatalytic materials and improving electrochemical energy conversion devices.

Nitrogen‐doped porous carbon (NPC) is a commonly used carbon material that can be combined with cobalt (Co) to enhance the activity and stability of water splitting. Li et al.^[26]^ calcined a mixture of Co(NO_3_)_2_ ⋅ 6H_2_O and ChCl/urea/glucose acid DES. During the calcination process, the DES formed NPC, successfully prepared Co nanoparticles/NPC (Co@NPC). The DES not only acted as a soft template to facilitate the formation of Co nanoclusters with well‐defined hierarchical structures, but it also resulted in a high and uniform nitrogen content after carbonization. This “one‐pot” calcination approach was a favorable catalyst preparation strategy due to its simplicity and environmentally friendly nature. Experimental results demonstrated that the synergistic effect between Co and NPC effectively enhanced the activity and stability of water splitting. This composite catalyst not only exhibited excellent HER performance (*η*_10_=215 under acidic conditions; *η*_10_=274 under alkaline conditions), but also demonstrated remarkable water splitting performance (potential of 1.74 V at a current density of 10 mA cm^−2^) and glucose decomposition performance (potential of 1.56 V at a current density of 10 mA cm^−2^).

The combination of cobalt with other metals to form alloys offers the ability to regulate the electronic structure and surface properties of cobalt, thereby altering the structure of reaction intermediates and promoting charge transfer and reaction rates. This leads to improved conductivity and loading performance, resulting in enhanced kinetics and selectivity for water splitting and overall electrocatalytic performance. Additionally, pure Co may suffer from deactivation and corrosion issues in certain electrocatalytic reactions. By introducing other metals into cobalt alloys, the stability and durability of the material can be improved, reducing cobalt dissolution and surface oxidation, and prolonging the catalyst‘s lifespan.[^27^, ^28^] This makes cobalt alloys an attractive option for catalytic applications. The high solubility of DES makes it a favorable medium for preparing cobalt alloys. For example, using ChCl/ethylene glycol (EG) DES as electrolyte dissolved with Ni, Co, and Sn ions, a Ni−Co−Sn alloy could be synthesized in one step without the addition of other substances.^[28]^ Similarly, The electrodeposition of Fe−Co was carried out using ChCl/urea DES.^[29]^ This method avoids interference from other factors and yields a desirable alloy composition. These results show that the utilization of cobalt alloys through the combination with other metals and the use of DES as a synthesis medium holds great promise for improving the electrocatalytic performance, stability, and durability of cobalt‐based catalysts in various applications. The use of DES provides a new avenue for creating novel catalysts that are not only superior in performance but also feature a more streamlined and greener synthesis process, marking an important advancement for the development of sustainable energy technologies. Future research can explore a wider variety of DES and metal combinations to discover more cobalt alloys with exceptional performance, which is crucial for advancing the development of clean energy technologies.

**Figure 2:**
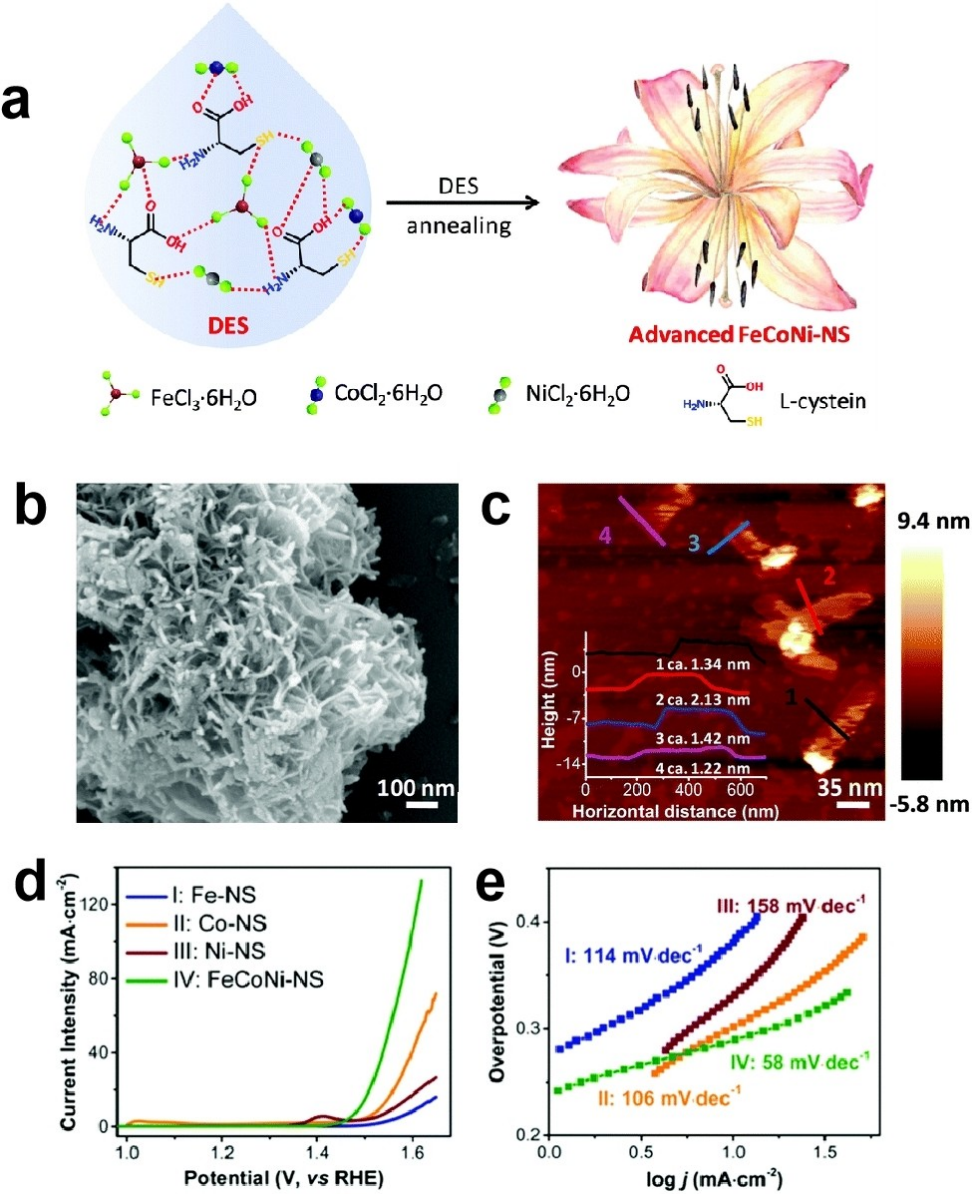
Illustration of the formation of N,S‐NiCoFe Alloy (a), SEM image of N,S‐NiCoFe alloy (b) and AFM image (c), LSV curves of N,S‐NiCoFe alloy and other comparative samples (d), and tafel slopes (e). Reprinted with permission from ref. [30], copyright 2019 RSC.

FeCl_3_ ⋅ 6H_2_O, CoCl_2_ ⋅ 6H_2_O and NiCl_2_ ⋅ 6H_2_O have similar properties. By mixing these three compounds in certain proportions, a hydrogen bond acceptor was obtained. Then the HBA was combined with L‐cysteine to form a novel DES. This DES can be directly calcined in a one‐step process to obtain N,S‐NiCoFe alloy.^[30]^ In this reaction, the DES serves multiple roles: solvent, template, coordinating agent, metal sources, N source, and S source (Figure 2a). The template effect of DES not only allows the catalyst to have a porous structure and high specific surface area but also promotes the uniform distribution and coordination of Ni, Co, and Fe (Figure 2b and c). The introduction of Fe (20 %–40 %) can stabilize the high oxidation states of other metals and significantly reduce the overpotential of the catalyst. In addition, research has demonstrated a significant interaction between Ni and OH^−^, which is expected to substantially decrease the Tafel slope. The effective integration of Fe, Ni, and Co components into a single material system can significantly enhance the catalytic performance. The prepared N,S‐NiCoFe alloy possessed a unique structure with multi‐metallic sites, high specific surface area, and a porous structure. It exhibited excellent OER performance under alkaline conditions (*η*_10_=251 mV, Tafel slope=58 mV dec^−1^) (Figure 2d and e). The stability of N,S‐NiCoFe was examined by multicurrentstep chronopotentiometry and multipotential‐step chronoamperometry (CA) experiments. After a 10‐hour CA test at 1.54 V, the current density remained almost unchanged (less than 2 %), achieving a 98 % Faraday efficiency. The morphology remained almost unchanged, indicating structural stability. Compared to other NiCo catalysts, the prepared N,S‐NiCoFe exhibits excellent activity, such as, NiCo_2_O_4_ (*η*_10_=320 mV, Tafel slope=61 mV dec^−1^),^[31]^ CoNi (*η*_10_=420 mV, Tafel slope=98.7 mV dec^−1^).^[32]^ The doping of iron can greatly enhance the catalytic activity. Iron atoms have strong redox capabilities and play a crucial role in catalytic reactions, especially in redox reactions.^[33]^ Iron atoms may act as electron donors or acceptors, thereby enhancing the catalytic activity of the catalyst.^[34]^ This method has the advantages of simplicity, efficiency, and scalability, while minimizing side reactions and high conversion rates, in line with the principles of atom economy. Additionally, it does not require tedious post‐processing steps and has the characteristics of being environmentally friendly, simple, and low‐energy consumption, making it an effective strategy in the pursuit of green synthesis technologies.

**Figure 3:**
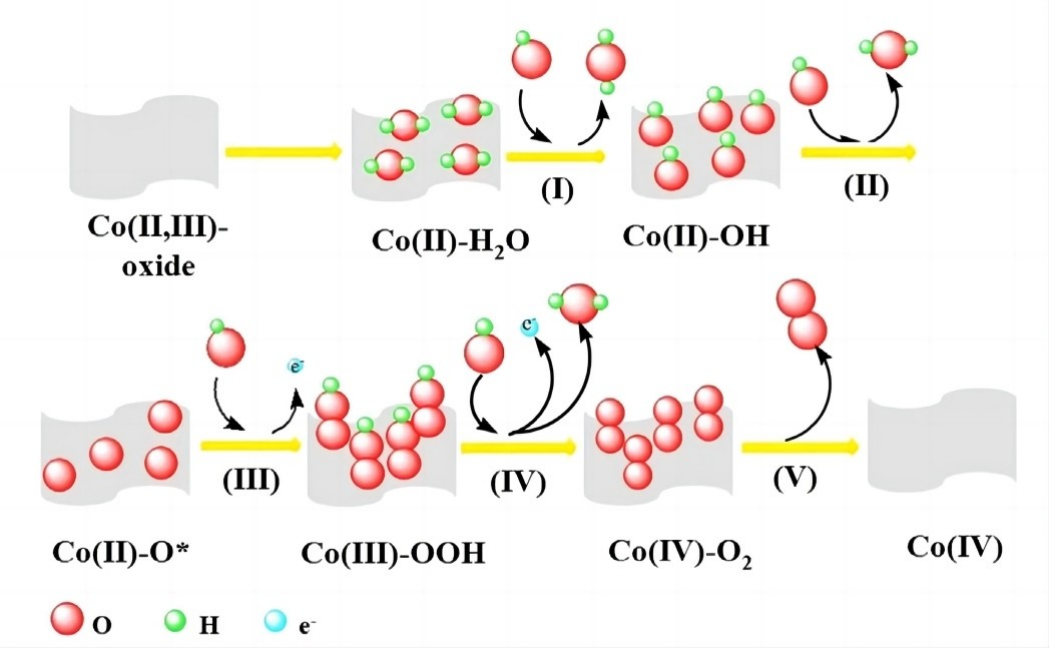
The possible electrocatalytic mechanism of Co‐based oxides for the OER. Reprinted with permission from ref. [35], copyright 2018 wiley.

Cobalt oxide is known for its low cost and high corrosion resistance. Studies have found that H_2_O molecules can be adsorbed onto the surface of cobalt oxide through hydrogen bonding or active sites on the oxide surface during water splitting process. Following adsorption, the water molecules are activated, resulting in the formation of active Co−O centers during the OER process. This activation enhances the conductivity of CoO_x_ and facilitates the efficient adsorption of OER intermediates such as OH* and OOH* (Figure 3).^[35]^ These intermediates play crucial roles in the overall water splitting process. By facilitating the adsorption and subsequent reactions of these intermediates, the activated CoO_x_ surface contributes to improved performance and efficiency in the OER process.

Our research group^[36]^ obtained ordered CoO nanosheets by calcining a solution containing Co(acac)_3_ in 1‐butyl‐3‐methylimidazolium bis(trifluoromethylsulfonyl)imide ([BMIM]Tf_2_N). According to time‐dependent studies, an in‐depth analysis of the growth process of CoO nanosheets has been conducted, yielding intriguing observations. It was found that CoO particles formed and aggregated together, with [BMIM]Tf_2_N adsorbed on the nanoparticles in the initial stage. The [BMIM^]+^ cations could self‐assemble into polymer supramolecular structures, which served as templates to aid in the formation of sheet‐like structures of CoO nanosheets. The presence of IL played a crucial role in regulating the aggregation behavior and ordering of the CoO nanosheets. The prepared cobalt oxide nanosheets exhibited excellent OER performance in 1 M KOH, with a low overpotential (*η*_10_=320 mV) and a small Tafel slope (70 mV dec^−1^). These impressive electrocatalytic properties highlight the potential of the prepared CoO nanosheets for efficient water splitting applications.

**Figure 4:**
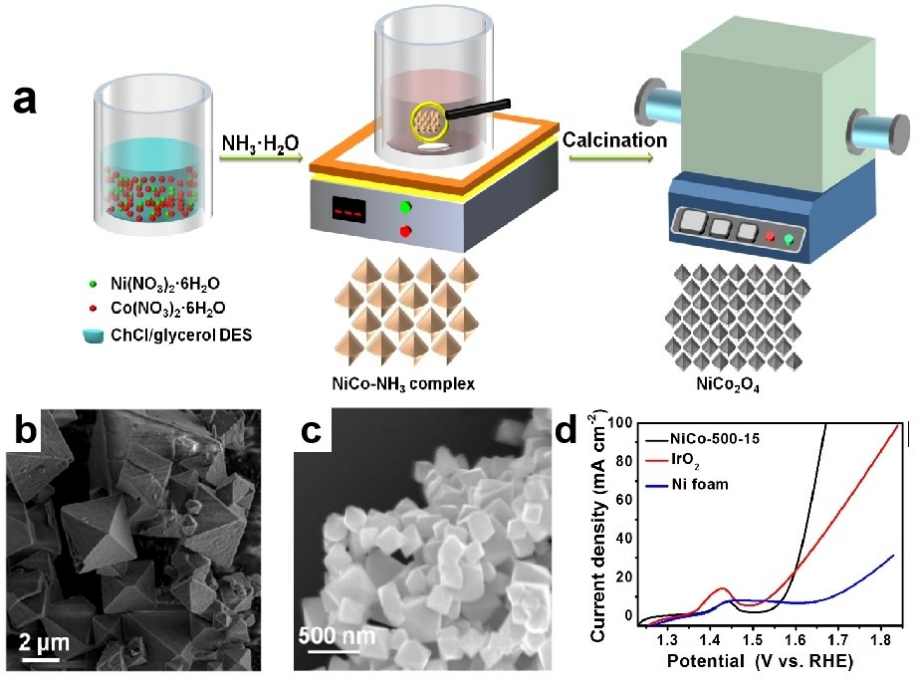
Schematic illustration of the synthesis process of NiCo_2_O_4_ (a), SEM images of NiCo‐NH_3_ composite (b) and NiCo_2_O_4_ (c), polarization curves of NiCo_2_O_4_ and other comparative materials (d). Reprinted with permission from ref. [31], copyright 2022 Elsevier.

Spinel is a typical cubic close‐packed structure, characterized by a tight lattice arrangement, low cost, adjustable composition, controllable morphology and structure. Cobalt oxide spinel is a commonly used catalyst, particularly playing a critical role in the process of electrolytic water splitting.[^31^, ^37^, ^38^] Our research group^[31]^ has developed a method for the preparation of octahedral NiCo_2_O_4_ using a two‐step process involving DES. Ammonia, CoCl_2_, and NiCl_2_ were added to ChCl/ glycerol DES. The DES assisted in obtaining the easily hydrolyzable NiCo−NH_3_ complex at room temperature during the first step. This avoided the harsh reaction conditions in aqueous phase that require low temperature and the presence of hydrolysis inhibitors. In the second step, the NiCo−NH_3_ complex was subjected to calcination, which only taked 15 min to achieve complete oxidation, resulting in the formation of NiCo_2_O_4_ with a unique octahedral morphology (Figure 4a–c). During the investigation of the influence of calcination temperature and time on the morphology and composition of the pyrolysis products, we discovered that the selected DES not only promoted the formation of precursors and oxides but also could regulate the morphology of intermediates and products. The synergistic effect of Ni and Co and the unique octahedral structure of the prepared NiCo_2_O_4_ exhibited excellent OER performance and exceptional stability. In a 1.0 M KOH electrolyte, the *η*_10_ and Tafel slope of NiCo_2_O_4_ was 320 mV and 67 mV dec^−1^, respectively (Figure 4d). The NiCo_2_O_4_ demonstrated remarkable stability. Its cyclic voltammetry results indicated that the curve was nearly identical to the initial condition following 2000 cycles. Additionally, the catalyst system sustained stability for 34 hours at a current density of 10 mA cm^−2^. The prepared NiCo_2_O_4_ exhibits higher catalytic activity than IrO_2_ in the OER during water splitting due to its unique electronic structure, good stability, cost‐effectiveness, conductivity, and potential synergistic effects. However, the specific catalytic mechanism may involve more complex factors that require further exploration through detailed experiments and theoretical calculations. In another study by Thorat et al.,^[38]^ octahedral‐shaped CoV_2_O_6_ was successfully synthesized using ChCl/glyceric acid DES by introducing cobalt salt and vanadium salt and reacting them at a lower temperature of 500 °C for 2 hours. In comparison, without utilizing DES, it is necessary to pyrolyze for 40 hours at a higher temperature of 720 °C to obtain CoV_2_O_6_. This work demonstrated that DES not only could regulate the morphology of CoV_2_O_6_ to exhibit a regular octahedral shape but also significantly reduced the reaction temperature and shortened the reaction time. The obtained octahedral CoV_2_O_6_ showed excellent catalytic activity in the OER with an *η*_10_ of 324 mV.

### 2.2 Cobalt‐Based Hydroxide Catalysts Based on ILs and DESs

Research has found that under alkaline conditions, the surface of transition metal catalysts can easily be transformed into hydroxides or hydroxyoxides during the oxygen evolution reaction.^[39]^ This discovery has sparked interest in exploring the catalytic properties of hydroxide catalysts. By rational design of catalyst structures, controlling surface active sites, and enhancing electron transfer capability, the efficiency and stability of hydroxide catalysts can be improved, thereby promoting research and applications in related fields.

Under alkaline conditions, 1‐butyl‐3‐methylimidazolium tetrafluoroborate ([BMIM]BF_4_) could be utilized as the reaction medium to efficiently synthesize single‐metal (Co, Ni, Fe) and bimetallic (FeCo, NiCo, NiFe) hydroxides.^[32]^ IL, as a solvent, exhibited excellent solubility and allowed for control over reaction conditions, composition, and structure. This IL not only acted as a soft template for the formation of hydroxide nanospheres but also lowered the free energy, restricting the growth of specific crystal facets and reducing the crystallinity of the products. This resulted in an increased surface area of the catalyst, enhancing its catalytic performance. All the hydroxides prepared in this work exhibited excellent OER performance. Taking CoFe hydroxide and CoNi hydroxide as examples, at a current density of 10 mA cm^−2^, the overpotentials for CoFe hydroxide and CoNi hydroxide were 350 mV and 420 mV, respectively. This work demonstrates the powerful capability of ILs in the preparation of highly efficient electrocatalysts, especially by tuning the reaction conditions to optimize the morphology and structure of the catalysts, thereby enhancing their performance. Moreover, the environmentally friendly and recyclable characteristics of ILs make them a green alternative to traditional organic solvents. Although the cost of synthesizing ILs is relatively high, their reusability and high efficiency may offset this disadvantage, making them still attractive for large‐scale industrial applications.

Layered double hydroxides (LDHs) are a special class of hydroxides, and the interlayer spacing plays a crucial role in their electrocatalytic performance. Increasing the interlayer spacing can enhance the chances of contact between metal ions and water molecules, facilitating the formation of more catalytically active sites. However, conventional methods for synthesizing LDH materials with large interlayer spacing typically involve a laborious two‐step process of precursor synthesis and subsequent ion exchange. This process is time‐consuming, often taking several hours, days, or even weeks, and requires stringent reaction conditions such as inert gas protection. Tu et al.^[40]^ developed a water injection method to synthesize CoFe LDH with a significantly enlarged interlayer spacing. In this method, an appropriate amount of CoCl_2_ ⋅ 6H_2_O and FeCl_3_ ⋅ 6H_2_O was added to a ChCl/urea DES. By gradually injecting small amounts of water into the solvent under heating conditions, urea hydrolyzed in the presence of water, generating abundant OH^−^ ions. These ions reacted with Co^2+^ and Fe^3+^ ions, resulting in the formation of Co(OH)_2_ and Fe(OH)_3_, and ultimately leading to the formation of LDH. During the reaction process, if the nucleation rate was slow or continues for an extended period, it could result in the formation of unevenly sized crystal particles. Rapid water injection quickly diluted the concentration of reactants in the reaction solution and provided a larger volume of solvent, thereby slowing down or preventing further continuous nucleation from occurring. This approach could promote the uniform formation of crystal particles in a relatively short period, resulting in a larger interlayer spacing. X‐ray diffraction (XRD) analysis indicated that the maximum interlayer spacing of this LDH can reach 11.3 Å. Fourier‐transform infrared (FTIR) spectroscopy revealed that the intercalated molecules in CoFe LDH were DES decomposition products, including acetaldehyde, ethanol, urea condensate, CO_3_^2−^, and Cl^−^. Owing to its large surface area, this compound exhibited outstanding catalytic performance in water splitting.

## 3 Summary and Outlook

This review focuses on the utilization of IL and DES in the preparation of cobalt and its oxides/hydroxides, which has achieved remarkable results in the field of electrolysis for water splitting. IL and DES serve various functions, acting as solvents, templates, and reactants in the synthesis process. They can effectively dissolve cobalt salts and other precursors, providing a homogeneous reaction environment that enhances the controllability and selectivity of the reactions. As a result, the performance of the obtained catalysts is significantly improved.[^40^, ^42^]

While IL‐ and DES‐induced cobalt‐based nanomaterials have shown promising potential for applications in water splitting, their practical use still requires further investigation and exploration:

1. Further optimization of IL and DES systems: The IL and DES systems is crucial for achieving efficient catalyst preparation. By adjusting the composition of ILs and DESs, selecting suitable cations and anions or HBDs and HBAs, as well as adding other functional components, better solubility, stability, and controllability can be achieved.
2. Sustainability: When designing IL and DES systems, it is important to not only follow the principles of green chemistry by selecting non‐toxic and renewable components but also to further optimize reaction conditions to reduce energy consumption and waste generation.
3. Achieving controllable synthesis: A deep understanding of the mechanism of ILs and DESs in the preparation of cobalt materials is required, including solvent‐metal interactions, solvent‐solvent interactions, and reaction mechanisms between solvents and precursors. This will help designing and controlling the preparation process, ultimately improving material performance.[^43^, ^44^, ^45^]

## Conflict of Interests

Authors declare no conflict of interest.

4

## Biographical Information

*Dr. Chenyun Zhang graduated from the School of Chemistry and Chemical Engineering at Shandong University and is currently employed at Wuxi Vocational Institute of Arts & Technology. Her research expertise lies in the preparation of water splitting catalysts using ionic liquids or deep eutectic solvents*.

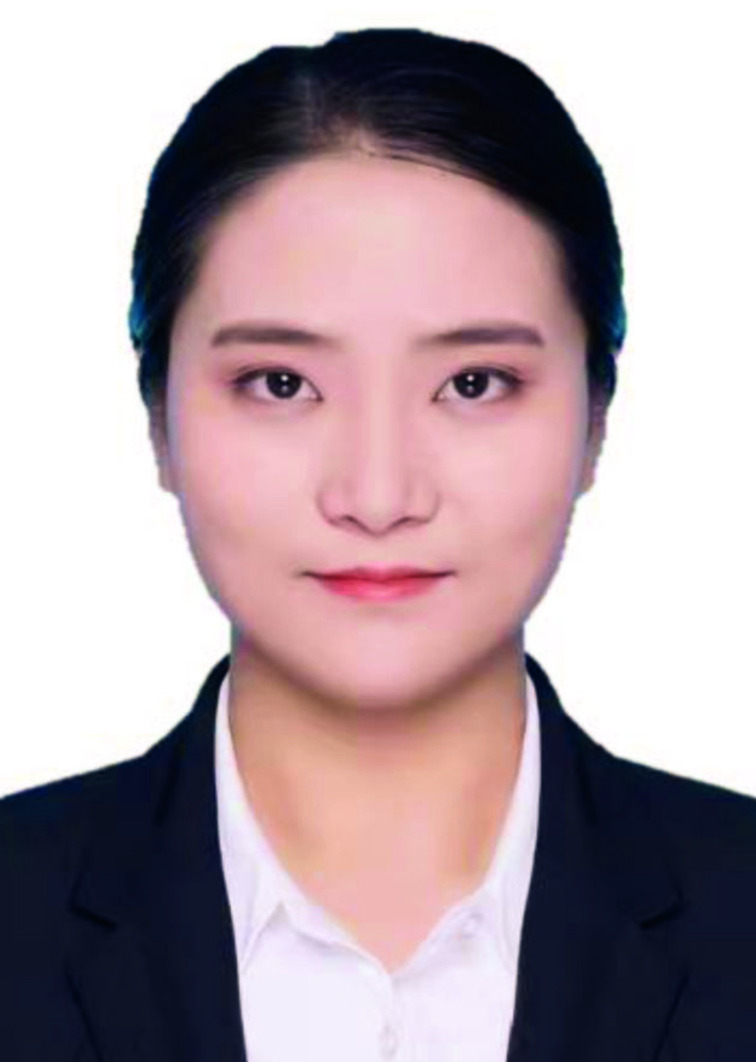

## Biographical Information

*Jie Wang is employed at Kaishi Faurecia Aftertreatment Control Technologies Co., Ltd, where he serves as the head of the Engineering and Technology Development Department. He is proficient in the field of new energy. He possesses extensive professional knowledge and practical experience in new energy technologies*.

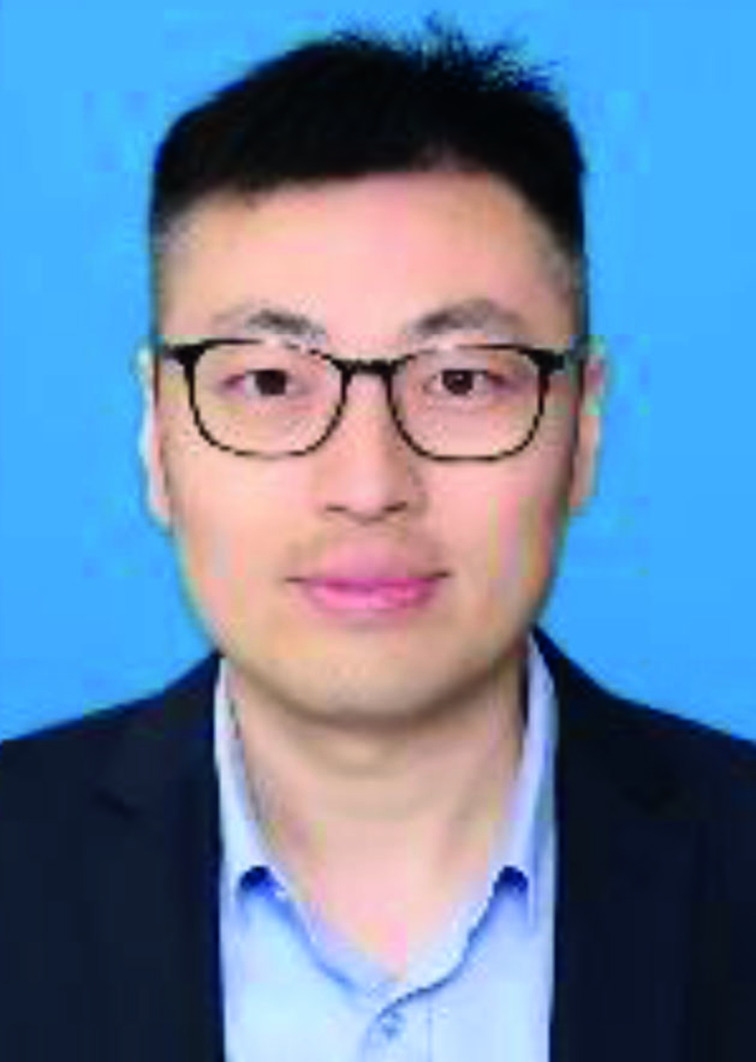

## Biographical Information

*Dr. Jianjiao Jin is employed at Shazhou Professional Institute of Technology. He has extensive teaching and research experience, with in‐depth research and understanding in the field of new energy technologies, particularly in the area of power battery recycling*.

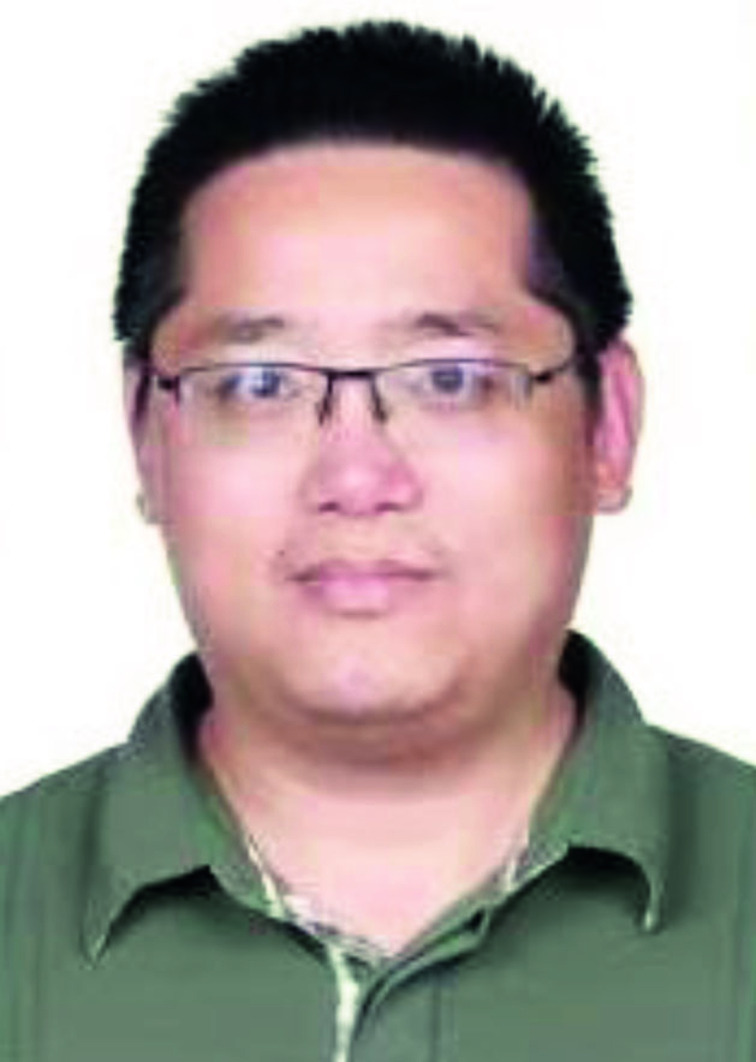

## Biographical Information

*Jiahao Wang holds a Master's degree in Environmental Engineering. He is currently employed at Wuxi Vocational Institute of Arts & Technology. He has participated in the National Science and Technology Support Program project: Zero Discharge Comprehensive Utilization of 10,000 Tons of Concentrated Seawater*.

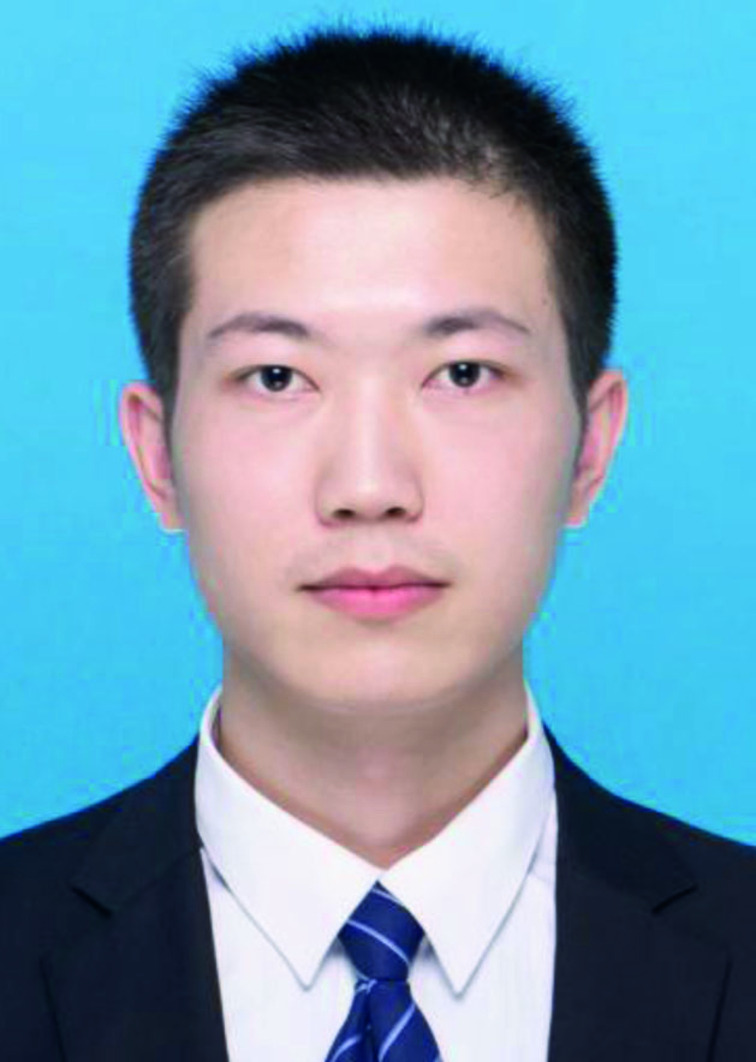

## Biographical Information

*Dr. Te Bai is employed at Wuxi Vocational College of Science and Technology. He has published two high‐quality research papers in the prestigious international chemistry journal “Angewandte Chemie”, reflecting his outstanding professional achievements in the field of chemistry*.

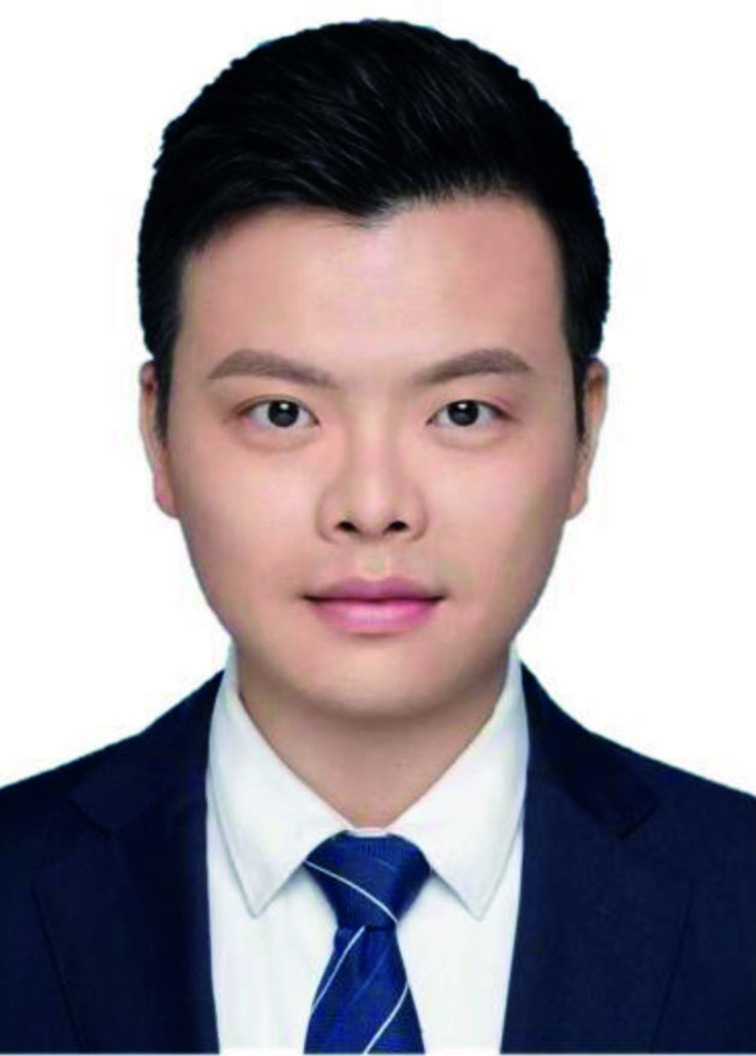

## Biographical Information

*Dr. Jiacheng Xu graduated from the School of Materials Science and Engineering at Changzhou University and currently serves as a full‐time teacher at the Ceramic Institute of Wuxi Vocational Institute of Arts & Technology. He has published over 10 SCI papers*.

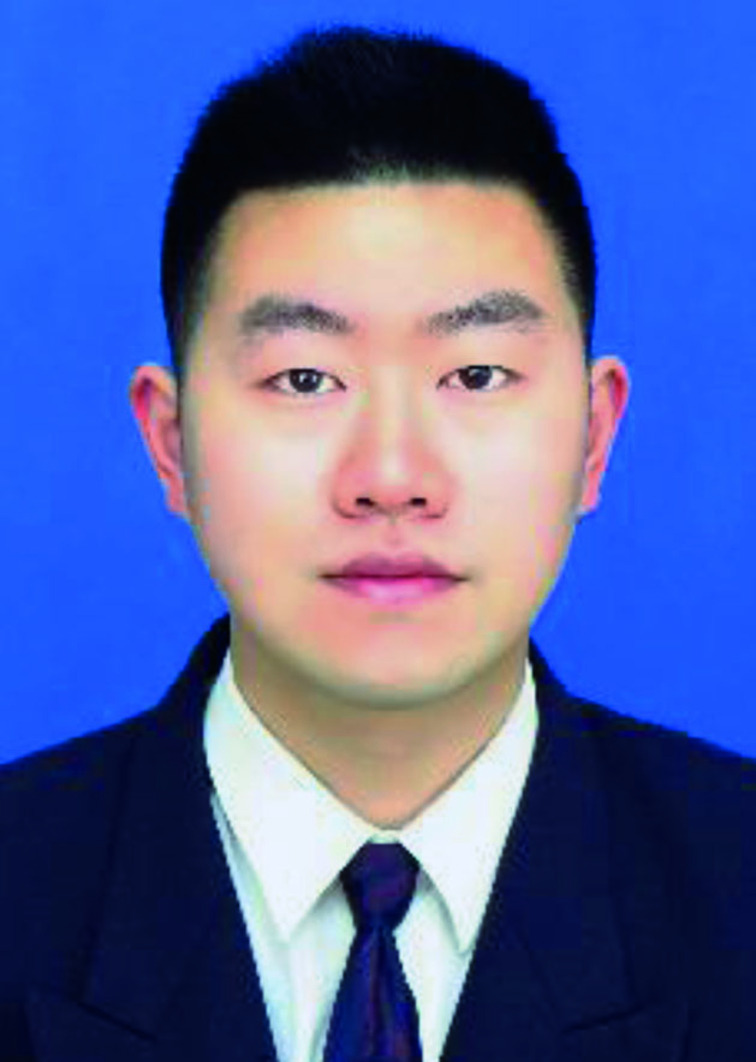

## Biographical Information

*Dr. Shun Wang currently serves as a full‐time teacher at the Ceramic Institute of Wuxi Vocational Institute of Arts & Technology. His primary research interests include solid oxide fuel cells, ceramic tape casting preparation, and corrosion of refractory materials by lithium electrodes. Dr. Wang has published 7 SCI papers*.

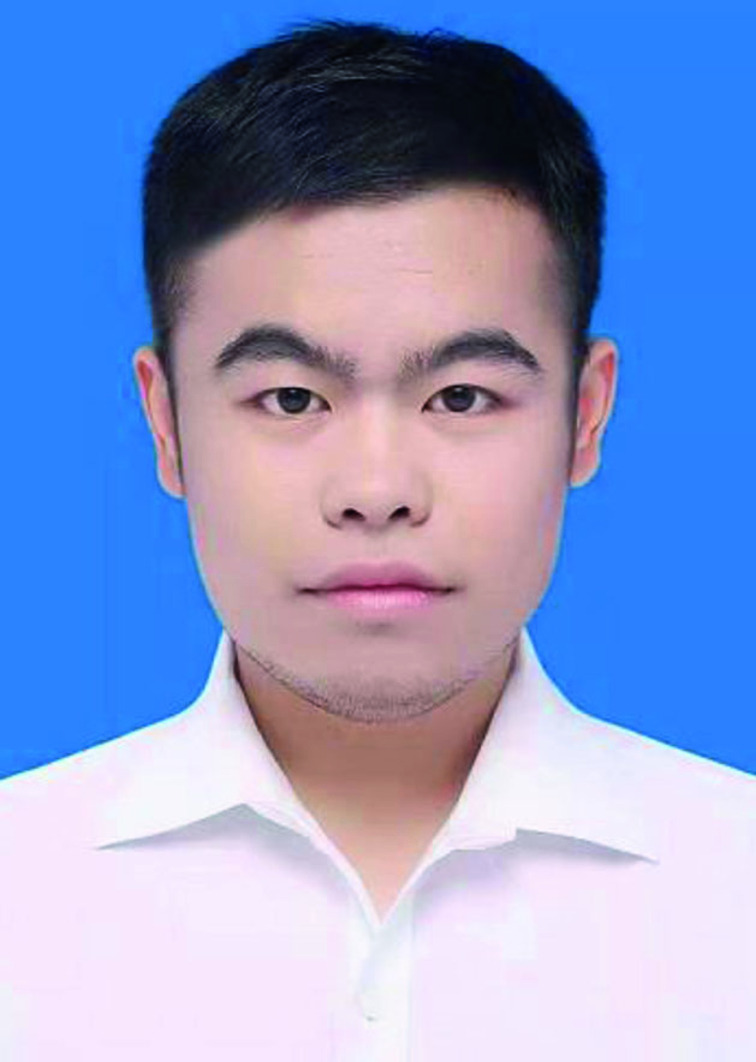

## Biographical Information

*Professor Lihua Xu graduated from the College of Chemical Engineering at Guizhou University. Currently, he serves as the Director of the Ceramic Technology Teaching and Research Office at the Ceramic Institute of Wuxi Vocational Institute of Arts & Technology, as well as an official judge for national vocational skills competitions and an assessor for national vocational skill appraisals*.

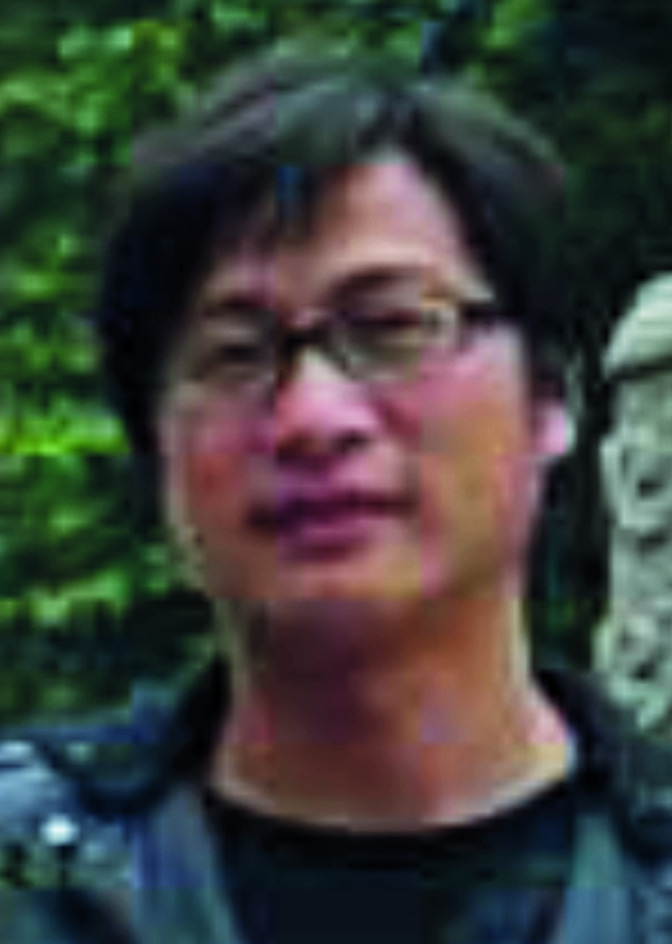

## Biographical Information

*Dr. Jing Zhang holds the position of associate professor and full‐time teacher at the Ceramic Institute of Wuxi Vocational Institute of Arts & Technology. In terms of scientific research, her focus areas include ceramic materials, inorganic non‐metallic materials, and microwave absorption materials, with more than 10 high‐level papers published*.

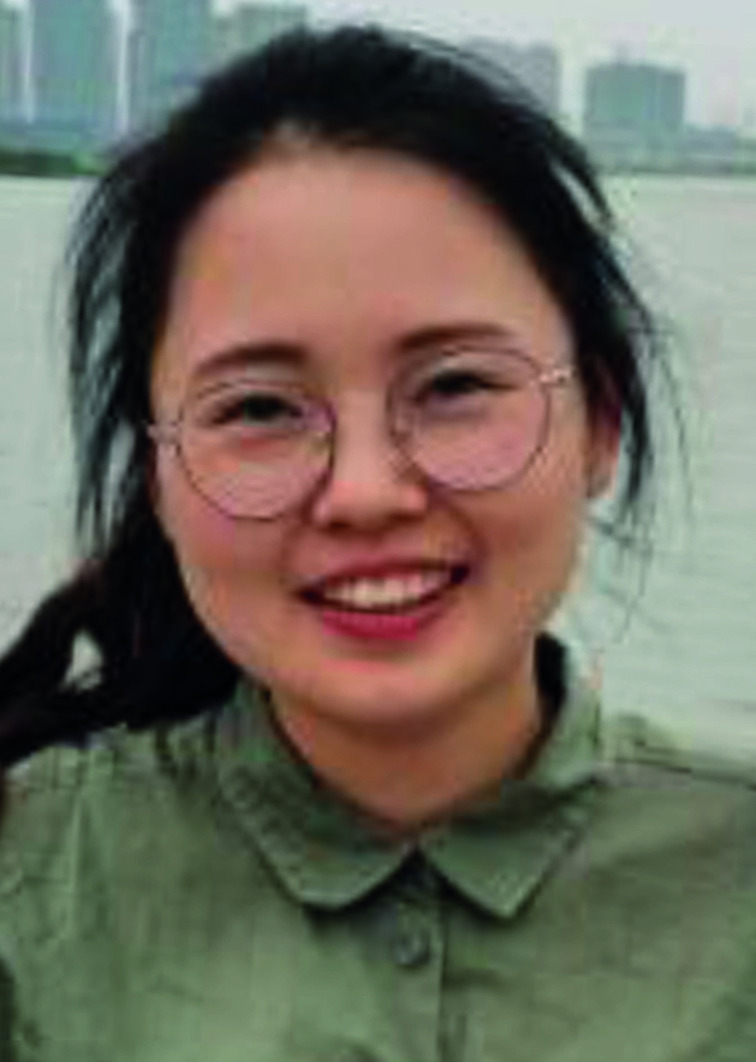

## Data Availability

All data analysed during this study are included in this published article.
